# *Candidatus* Neoehrlichia sp. in an Austrian fox is distinct from *Candidatus* Neoehrlichia mikurensis, but closer related to *Candidatus* Neoehrlichia lotoris

**DOI:** 10.1186/s13071-015-1163-0

**Published:** 2015-10-15

**Authors:** Adnan Hodžić, Rita Cézanne, Georg Gerhard Duscher, Josef Harl, Walter Glawischnig, Hans-Peter Fuehrer

**Affiliations:** Department of Pathobiology, Institute of Parasitology, University of Veterinary Medicine Vienna, Veterinärplatz 1, 1210 Vienna, Austria; Institute for Veterinary Disease Control, Austrian Agency for Health and Food Safety, Innsbruck, Austria

**Keywords:** *Candidatus* Neoehrlichia sp, 16S rRNA, *GroEL*, Red fox, Austria, Phylogenetic analysis

## Abstract

**Background:**

*Candidatus* Neoehrlichia came under the focus of recent research in terms of human and pet relevance. *Candidatus* Neoehrlichia mikurensis seems to be relatively abundant in animals and humans from Central European countries, whereas *Candidatus* Neoehrlichia lotoris was found solely in raccoons from the USA.

**Findings:**

Spleen samples from a total of 164 red foxes, originating from two western provinces in Austria (Tyrol and Vorarlberg), were collected and examined for the presence of tick-borne bacteria of the family *Anaplasmataceae* by PCR and sequencing. In a fox sample originating from Vorarlberg *Candidatus* Neoehrlichia sp. was found, which is genetically (16S rRNA, *groEL*) closely related to *Candidatus* Neoehrlichia lotoris but clearly distinct from *Candidatus* Neoehrlichia mikurensis.

**Conclusions:**

The present study revealed, for the first time, the occurrence of *Candidatus* Neoehrlichia sp. in a red fox worldwide. A continuing screening of wild carnivores, especially foxes, and ticks for this potential pathogen is required to evaluate the actual occurrence and distribution of these bacteria. Further research is needed to elucidate the relationships of Neoehrlichia, as well as their reservoir and impact on wildlife, pets and humans.

## Findings

*Candidatus* Neoehrlichia came under the focus of recent research in terms of human and pet relevance [1]. The coccoid Gram-negative bacteria *Candidatus* Neoehrlichia mikurensis (CNM) and *Candidatus* Neoehrlichia lotoris (CNL) are supposed to be mainly associated with rodents and raccoons, respectively [1, 2]. Moreover, CNM was found in humans, dogs, hedgehogs, shrews, bears, badgers, chamois, mouflons and ticks collected from various wild animals [1, 3–5]. CNL was solely found in raccoons in the USA [2, 6] and trials to experimentally infect laboratory mice, rats or rabbits failed [7]. The vectors of CNM are supposed to be mainly *Ixodes ricinus* and other *Ixodes* species, but the pathogen was also detected in *Dermacentor reticulatus*, *Rhipicephalus sanguineus*, *Haemaphysalis concinna* and *H. leachi* [1]. For CNL *Ixodes* spp. are assumed to be potential vectors [7], but further research is needed to confirm the vector competence of different tick species. Until now several studies, mainly on the *groEL* gene, indicated a considerable genetic variation within CNM in Europe [8], whereas for CNL only a single variant has been described yet [2].

In the year 2014 spleen samples were collected from 164 foxes in two western provinces of Austria (Tyrol and Vorarlberg). DNA was extracted from ~20 mg of spleen tissue using the DNeasy Blood & Tissue Kit (QIAGEN, Netherlands) according to the manufacturer’s instructions. All samples were screened for *Anaplasma* spp., *Ehrlichia* spp. and *Candidatus* Neoehrlichia using the *Anaplasmataceae*-specific primers EHR16SD and EHR16SR, which amplify a ~345 bp section of the 16S rRNA (*16S*) [9]. PCR analysis of the spleen of one female fox (FU98) originating from Feldkirch (Vorarlberg) gave a positive signal. For a more integral approach we designed primers amplifying a longer (~1,053 bp) fragment of the *16S* and a 806 bp section of the *groEL* gene. The DNA fragments were amplified with the GoTaq® G2 Polymerase (Promega, USA). The PCR started with 2 min at 95 °C, followed by 35 cycles with 1 min at 95 °C, 1 min at the particular annealing temperature (Table 1), 1 min at 72 °C, and a final extension for 5 min at 72 °C.

**Table 1:** PCR conditions for identification of *Candidatus* Neoehrlichia used in this study

Specifity	Genetic marker	Sequences of primer (5’- 3’)	Annealing temperature (°C)	Amplicon size (bp)	References
*Anaplasmataceae*	16S	EHR16SD: GGT ACC YAC AGA AGA AGT CC	54	345	[9]
		EHR16SR: TAG CAC TCA TCG TTT ACA GC			
*Candidatus* Neoehrlichia	*groEL*	NeoeGroELFw: CAG GTG AAG CAC TAG ATA AGT CCA	54	806	This study
		NeoeGroELRv: ACA GCA GCA ACA TGC AAT CCA			
*Candidatus* Neoehrlichia	16S	16SCNM_for: GTG GCA GAC GGG TGA GTA AT	60	1,053	This study
		16SCNM_rev: TGC AGC ACC TGT GTA AGG TC			

A phylogenetic tree was constructed with the combined *16S* [KT833357] and *groEL* [KT833358] sequences of the sample FU98 and *Candidatus* Neoehrlichia sequences published at the NCBI data base (www.ncbi.nlm.nih.gov). *16S* and *groEL* sequences originating from the same hosts were published only for nine samples of CNM and one of CNL, respectively. For outgroup comparison, *16S* and *groEL* sequences were extracted from the complete genome of *Ehrlichia chaffensis* [CP007479]. The two sequence sections were aligned separately with MAFFT v.7.215 [10], resulting in alignments of 884 bp and 686 bp for *16S* and *groEL*, respectively. The two alignments were concatenated and a model test was performed with JModeltest v.2.1.5 [11]. A Maximum Likelihood (ML) bootstrap tree (1000 replicates) was calculated with MEGA6 v.6.06 [12] with the suggested substitution model GTR + G + I and Subtree-Pruning-Regrafting as heuristic method.

Phylogenetic networks were calculated with the *16S* and *groEL* sequences of the newly found *Candidatus* Neoehrlichia sp. (FU98) and published data. BLAST searches for *Candidatus* Neoehrlichia were performed at the NCBI data base with the *16S* and *groEL* sequences. The sequences of both data sets were aligned with MAFFT v.7.215 [10] and Median-Joining networks were calculated with Network v.4.6.0.0 (fluxus-engineering.com) applying the default settings. Genetic distances were calculated with MEGA6 v.6.06 [12] based on the *16S* and *groEL* alignments used for the phylogenetic networks. Mean *p*-distances were calculated between CNM and CNL and maximum *p*-distances were calculated within those taxa.

**Fig. 1:**
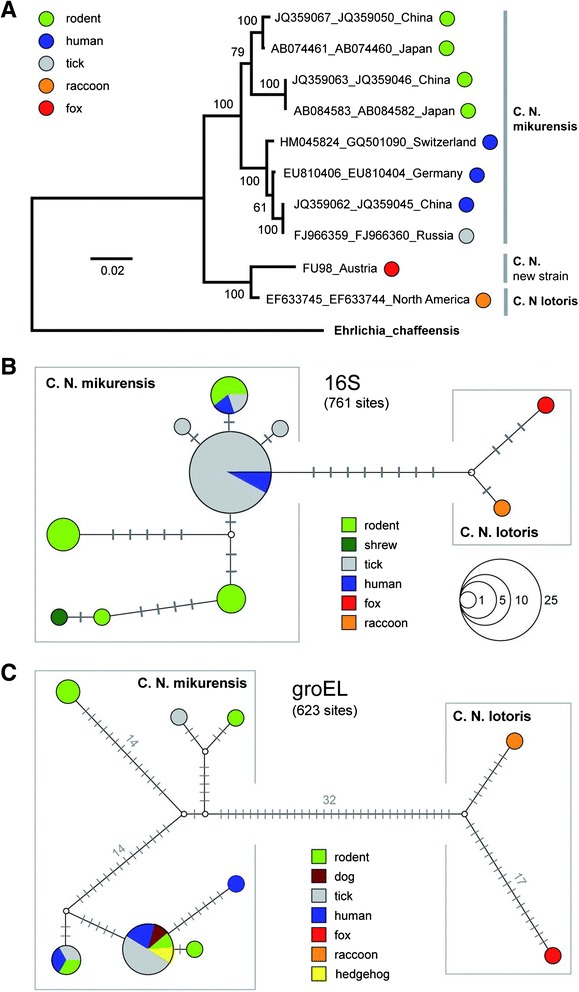
**a** Maximum Likelihood bootstrap tree with *16S* and *groEL* sequences of CNM and CNL. ML bootstrap values are indicated at the nodes. The tree is drawn to scale, with branch lengths measured in the number of substitutions per site. **b** Median-Joining network with *16S* sequences of *Candidatus* Neoehrlichia. The sizes of the nodes correspond to the number of haplotypes (right lower corner). Grey bars indicate the number of substitutions between haplotypes. **c** Median-Joining network with *groEL* sequences of *Candidatus* Neoehrlichia

The ML bootstrap tree calculated with the concatenated alignments of *16S* and *groEL* (1,570 bp) (Fig. 1a) shows two highly supported clades, the first with samples classified as CNM, the second containing the only CNL sample published yet, RAC413 [2], as well as the new *Candidatus* Neoehrlichia sp. (FU98) originated from a fox in the present study. The Median-Joining networks (Fig. 1b and c) both show well separated clades containing exclusively sequences of CNM and CNL. The maximum *p*-distances within CNL (= between FU98 and RAC413) are 0.5 % (*16S*) and 4.2 % (*groEL*), whereas the maximum *p*-distances within CNM are slightly higher with 1.2 % (*16S*) and 5.7 % (*groEL*). The mean genetic *p*-distances between CNM and CNL are 1.3 % (*16S*) and 8.9 % (*groEL*), and thus higher than the maximum intraspecific distances measured within the two taxa.

## Conclusions

This study reports the presence of *Candidatus* Neoehrlichia sp. in a red fox for the first time worldwide. The obtained sequences are considered as CNL in the present study because of the similarity with the strain RAC413, which was isolated from raccoons in the south-eastern USA. In the phylogenetic tree calculated with sections of the *16S* and *groEL* genes, the strains FU98 and RAC413 from well supported clade, clearly distinct from CNM. Genetic distances between FU98 and RAC413 are only slightly lower than those within CNM. However, the current data is not sufficient to explicitly state whether the new FU98 sequences belong to CNL or rather represents a new species of *Candidatus* Neoehrlichia. According to the national surveillance for the occurrence of raccoons and raccoon dogs, there is an oral report of a sighting in 2010 in this particular area and a proven evidence of raccoons ~15 km north of the investigation area in 2011 (Duscher T., person. comm.), although their abundance is sporadic. Nevertheless spill over from these raccoons cannot be excluded. However, investigations of free ranging Austrian raccoons are needed to trace the infection ways. Moreover, a continuing screening of wild carnivores, especially foxes, and ticks for this potential pathogen is required to see the actual occurrence and distribution of these bacteria. Further research is needed to elucidate the relationships of Neoehrlichia, as well as their reservoir and impact on wildlife, pets and humans.

### Ethical statement

All foxes were shot during routine hunting events under the restrictions of the game laws of Austria.
